# Prevalence of visual impairment in older people living with dementia and its impact: a scoping review

**DOI:** 10.1186/s12877-022-03581-8

**Published:** 2023-02-01

**Authors:** Wanyu Zhang, Timothy V. Roberts, Christopher J. Poulos, Fiona F. Stanaway

**Affiliations:** 1 School of Public Health, Faculty of Medicine and Health, The University of Sydney, Camperdown, NSW 2006 Australia; 2 Sydney Medical School, Faculty of Medicine and Health, The University of Sydney, Camperdown, Sydney, NSW 2006 Australia; 3 Department of Ophthalmology, Royal North Shore Hospital, Reserve Rd, St Leonards, NSW 2065 Australia; 4 Vision Eye Institute, Level 3, 270 Victoria Ave, Chatswood, Sydney, NSW 2067 Australia; 5 HammondCare, 4/207B Pacific Hwy, St Leonards, Sydney, NSW 2065 Australia; 6 School of Population Health, The University of NSW, Samuels Building, Samuel Terry Ave, Kensington, Sydney, NSW 2033 Australia

**Keywords:** Dementia, Elderly, Older adults, Alzheimer’s, Visual impairment, Sight loss, Glaucoma, Cataract, Sight loss, Age-related macular degeneration, Scoping review

## Abstract

**Background and objectives:**

Visual impairment (VI) and dementia both increase with age, and it is likely that many older people are living with both conditions. This scoping review aims to investigate the prevalence and types of VI among older people living with dementia, and the impact of VI on older people living with dementia and their caregivers.

**Methods:**

This scoping review used Arksey and O’Malley’s methodological framework. Studies in any setting involving people living with dementia and some assessment of either VI, eye diseases causing VI or the impact of VI were included.

**Results:**

Thirty-six studies were included. Thirty-one studies reported the prevalence of VI in older people living with dementia, while ten studies reported on impacts of VI on people living with dementia. Only one study reported on impacts on caregivers. The prevalence of VI or specific eye diseases among older people living with dementia ranged from 0.2 to 74%. The impacts of VI on older people living with dementia included increased use of hospital services, increased disability and dependency, reduced social engagement, negative emotions, increased abnormal behaviours, loss of hobbies, difficulty in using visual aids or memory aids, and greater Neuropsychiatric Inventory symptoms. And the impacts on caregivers included increased conflict and physical exhaustion.

**Conclusion:**

VI is common in older people living with dementia and is associated with negative impacts on those with dementia and their caregivers. However, heterogeneity between studies in terms of setting and method for assessing and defining VI make it difficult to compare findings among studies. Further research is needed, particularly assessing the impact on caregivers.

**Supplementary Information:**

The online version contains supplementary material available at 10.1186/s12877-022-03581-8.

## Introduction

Globally there were an estimated 43.8 million older people living with dementia in 2016 [1] and 173 million persons with severe or moderate visual impairment (VI) in 2010 [2]. Age is a risk factor for both conditions, meaning that the prevalence of both conditions increases with more advanced age. This also means that many older people are likely to be living with both conditions, with a likely increased impact on independence and quality of life.

As well as increasing the risk of harmful events such as falls, VI could increase the incidence of disorientation, confusion, inability to perform tasks of daily living, poor mental health and social isolation [3, 4].The presence of dementia in those with VI may hinder both the identification and management of vision impairment, leading to the exacerbation of both conditions [5]. It may also negatively impact on the self- management of chronic disease, leading to a higher utilization of healthcare services [6–8]. There is also an increased risk of family and carers experiencing feelings of exhaustion and depression [9]. Despite the expected increase in frequency of concurrent dementia and VI and the likely impact of concurrent VI on those living with dementia and their carers, there has been limited research describing the prevalence of VI in those living with dementia and its impact on quality of life and function in those living with dementia and their caregivers.

A previous scoping review by Bunn et al. explored the extent, range and nature of research in relation to dementia and comorbidity, with a specific focus on the comorbidities of diabetes, stroke and VI [10]. However, the literature search for this review was conducted in 2013 and only five studies that assessed the prevalence of VI were located, with four of the studies focusing on particular causes of VI such as glaucoma. In addition, there was minimal discussion of the impact of VI on those living with dementia apart from the impact on quality of care received. Finally, the impact of concurrent VI and dementia on caregivers was not assessed as part of the review. Our aim was to conduct a scoping review of the literature to identify and describe research about the prevalence and types of VI in older people living with dementia, and the impact of VI on older people living with dementia and their caregivers.

We aimed to answer three research questions: 1) What is the prevalence of VI in those living with dementia; 2) What is the impact of comorbid VI in those living with dementia; and 3) What is the impact of comorbid VI and dementia on caregivers of those living with dementia.

## Methods

This scoping review was conducted based on Arksey and O’Malley’s methodological framework [11]. A protocol for the study was developed and a comprehensive search conducted in electronic databases.

### Inclusion and exclusion criteria

We included studies involving people with any type of dementia in any setting with some measure of VI or eye diseases. In addition to studies on those living with dementia, studies of caregivers of people living with dementia and VI that measured the impact of combined dementia and VI on caregivers were included. We accepted any definition of VI including formally assessed visual acuity and self-reported VI. We also included studies that assessed the presence of common eye diseases in older people living with dementia such as age-related macular degeneration, glaucoma, cataract and diabetic retinopathy. Studies that examined visual symptoms or disorders of visual perception that were considered part of the dementia process, as opposed to a separate comorbidity, were excluded. We included all study types including cross-sectional, case-control, cohort, randomised controlled trials and qualitative studies in line with the broad scope of a scoping review.

### Search strategy

A comprehensive literature search strategy (see Additional file 1) was developed in partnership with a medical librarian. We searched for published and unpublished literature with no date or language restrictions. Literature in languages other than English were translated using google translate or with the assistance of native speakers (see acknowledgement). Searches were conducted in Ovid MEDLINE, Embase, PsycINFO, CINAHL, Scopus, Web of Science, Google Scholar and Open Grey. The search was conducted on 13th April, 2020.

### Selecting studies and charting the data

Two authors (WZ, FFS) screened titles and abstracts independently to select studies for inclusion. The full text of articles selected by either author in the initial screening stage were reviewed to select the final list of articles. Disagreements were resolved by discussion between WZ and FFS. Data were extracted independently by two authors (WZ, FFS) and checked for errors by comparing extracted data between both authors. Data extraction templates included information on authors, year of publication, country, study design, research question, inclusion/ exclusion criteria, representativeness, sample size, type of dementia, type of VI, how dementia/ VI were assessed, type of impact, how the impact was measured, size of effect and prevalence. Extracted data was reported narratively and summarized in tables. When a single study was published as several papers, these papers were grouped together and the one with the more complete data was considered the primary source. The quality of included studies was not formally assessed as this is a scoping review. However, data on study type, sample representativeness, sample size and the methods of diagnosing dementia and VI or other eye diseases was collected. We assessed the representativeness of study samples based on sampling methods and participation rates. Sampling methods considered representative included recruiting over a number of different sites to capture diverse populations in the community, or using insurance databases that have wide population coverage.

## Results

**Fig. 1:**
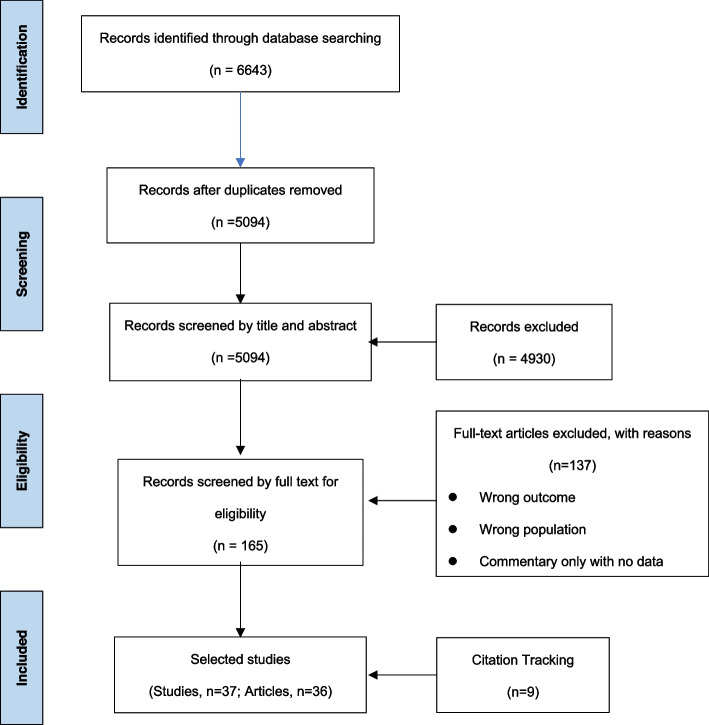
PRISMA Flow chart

A total of 5094 studies were identified after removal of duplicates (Fig. 1). After exclusion of non-relevant results by title and abstract screening, 165 articles were screened by full text and 37 studies (36 articles) included. Reasons for study exclusion included incorrect study population and no outcome data (no data on prevalence or impact of VI).

### Overview of study characteristics

Of the 37 studies included, 34 studies were quantitative studies, three studies were qualitative studies, and one study provided both quantitative and qualitative data. Thirty-one provided data on prevalence and ten provided data on the impact of VI on older people living with dementia (Table 1). Only one qualitative study and one quantitative study provided data on the impact of comorbid VI on caregivers. Most studies were from the United States of America (USA) (nine studies) [13, 19, 23, 25, 28, 35, 38, 41, 43] and the United Kingdom (UK) (ten studies) [12, 16, 17, 22, 26, 27, 37, 44, 47]. Three studies were from China [21, 31, 33] and one global study [40] reported data from low- and middle-income countries (Table 1). The majority of studies recruited samples from the community (59%). Eight studies (22%) recruited participants from nursing homes and seven (19%) were conducted in hospitals or other health-care settings.

**Table 1:** Study characteristics of all included studies

Study and Year	Country	Study type	Age (years)	Sex	Study sample selection and representativeness	N with dementia	Method of dementia diagnosis	Type of VI/eye disease	Method of VI/eye disease diagnosis
Abdullah,1965 [12]	UK	Not described	≥ 65	Male 26.5% Female 73.5%	3 patient groups - geriatric patients in London hospital; those of similar age seen on domiciliary visits; those attending a local social club. Moderately representative.	Unclear. 79 or 103	Not described	General VI Cataract	Ability to read or not Not described
Amjad, 2019 [13]	USA	Cross-sectional	≥65; Mean: 82.3 ± 8.1	Male 41.6% Female 59.4%	Community dwelling adults participating in 2011 NHATS (population- based nationally representative cohort of Medicare beneficiaries aged 65+), who died by 2015. Highly representative	542	Based on algorithm: clinician diagnosis of dementia or AD; AD-8 dementia screening interview of proxy respondents	VI	Based on interview questions including: ‘Uses corrective lenses or blind’, ‘Sees well enough to recognize person across street’, ‘Can watch TV across room’, ‘Reads newspaper print’
Bayer, 2002 [14]	Germany	Case-control	With glaucoma; Mean: 72.9 ± 10.6 Without glaucoma Mean: 71.4 ± 11.9	Male 35.7% Female 64.3%	4 nursing homes in Upper Bavaria, Germany. Low representativeness	112	Based on criteria for probable dementia based on NINCDS-ADRDA	Glaucoma	Visual field defects and/or optic disk cup-to-disk ratio of 0.8 or greater with an optic nerve head appearance consistent with glaucoma. Eye exams performed by one of the investigators.
Bayer, 2002 [15]	Germany	Case- control	With glaucoma; mean: 71.9 ± 11.6 Without glaucoma mean: 73.2 ± 12.3^a^	Male 38.8% Female 61.2%	2 nursing homes in Upper Bavaria, Germany. Low representativeness	49	Not described	Glaucoma	Visual field defects and/or optic disk cup-to-disk ratio of 0.8 or greater with an optic nerve head appearance consistent with glaucoma. Eye exams performed by two investigators.
Bennett, 2018 [16]	UK	Cohort study	Not described	Not described	Randomly sampled from primary care list in 3 areas of England. CFAS I, with an 80% response rate, CFAS II, with a 56% response rate. Highly representative.	83 in CFAS I and 277 in CFAS II.	Diagnosis via an algorithm	VI	Self-reported with no further details.
Bowen, 2016 [17]	UK	Cross-sectional	60–89	Male 37.9% Female 62.1%	Recruited from 20 NHS sites in six English regions. Includes both those in the community and nursing homes. Participation rate not reported but 100 withdrew (12.4%). Highly representative.	708 (VA measured in only 588)	Known diagnosis of dementia. Unclear how this data was obtained.	VI	Visual acuity (logMAR) worse than 6/12 or worse than 6/18 measured before and after refraction as assessed by an optometrist. Or Blindness – VA of < 3/60 in the better eye with presenting correction (ICD-10,12 categories 3–5) or visual field of no greater than 10 degrees in radius around central fixation.
								AMD	AMD was classified into dry and wet (neovascular) AMD and then graded as mild, moderate or severe. Based on medical records and optometrist exam.
								Cataract	Cataract sufficient to be graded on the TOC cataract grading scale. Based on medical records and optometrist exam.
								Diabetic Retinopathy	Based on medical records and optometrist exam
								Glaucoma	Based on medical records and optometrist exam
Bowen, 2016 [17]	UK	Qualitative	60–89	Male 50%; Female 50%	Purposive sampling from community setting and based on those who participated in the prevalence study above. Low representativeness.	36	Unclear how this data was obtained.	VI	Visual acuity (logMAR) worse than 6/12 or worse than 6/18 measured before and after refraction as assessed by an optometrist. Or Blindness – VA of < 3/60 in the better eye with presenting correction (ICD-10,12 categories 3–5) or visual field of no greater than 10 degrees in radius around central fixation.
								AMD	AMD was classified into dry and wet (neovascular) AMD and then graded as mild, moderate or severe. Based on medical records and optometrist exam.
								Cataract	Cataract sufficient to be graded on the TOC cataract grading scale. Based on medical records and optometrist exam.
								Diabetic Retinopathy	Based on medical records and optometrist exam
								Glaucoma	Based on medical records and optometrist exam
Carcenac, 2009 [18]	Canada	Cross-sectional	Not described	Not described	Nursing home setting. Only includes those who have died between April 2000 and April 2004. Not representative as only includes those who have died.	228	Not described. Extracted from clinical files of deceased patients.	AMD; Glaucoma	Based on clinical files of deceased patients.
Chandra, 1986 [19]	USA	Case-control	Mean: 80.1	Male 40.6% Female 59.4%	Community setting. Only includes those who have died (as dementia status is based on cause of death). Not representative.	7195	Listed as cause of death	Cataract Glaucoma Blindness	Listed on death certificate
Chriqui, 2017 [20]	Canada	Cross-sectional	≥ 65; Range 68 to 102; Mean: 87.2 ± 7.5	Male 26.7%; Female 73.3%	Residents of nursing homes. Populations of these facilities are representative of community nursing homes in the US. Response/completion rate of 50.8%. Moderately representative for nursing home setting.	150	Diagnosis recorded in medical chart.	VI	Distance VA lower than 6/12 (0.30 logMAR 20/40) in the better seeing eye as assessed by an optometrist.
Chung, 2015 [21]	Taiwan	Case-control	≥45; Mean 76.8 ± 9.6	Male 45.2% Female 54.8%	1,000,000 individuals randomly sampled from the Registry for Beneficiaries (*n* = 23.72 million) of the Taiwan National Health Insurance (NHI) program. Highly representative as most people in Taiwan are covered by the NHI.	7770	Diagnosis of dementia on claims records with ICD-9 codes. At least one diagnosis made by certified neurologist or psychiatrist.	Glaucoma	Based on ICD-9 codes in claims data.
Clague, 2017 [22]	UK	Cross-sectional	Mean: 82.6 ± 7.4;	Male 29.4% Female 70.6%	All registered patients who were alive and permanently registered in the Primary Care Clinical Informatics Unit with 314 general practices on 31 March 2007. Representative sample of the Scottish population. Highly representative.	10,528	Diagnosis from electronic medical records	Blindness or low vision	Identified open angle glaucoma cases by the principal diagnosis of ICD-9-CM codes 365.1, 365.10, or 365.11 in a medical claim during ambulatory care visits.
Deardorff, 2019 [23]	USA	Cross-sectional	≥65	Not described	Community-dwelling Medicare beneficiaries, enrolled in the MCBS between 1999 and 2006. Highly representative.	871	Self-report	VI	Based on question: How much trouble do you have with your vision? (no trouble, little trouble, or a lot of trouble). Subjects who reported “little trouble” or “a lot of trouble” were classified as having VI.
Frost, 2016 [24]	Australia	Case-control	Mean: 70.2 ± 9.0	Male 59% Female 41%	Community setting. Recruited from AIBL study in Western Australia (a study of over 2000 people with long-term follow up over 10 years). Moderately representative	22	NINCDS-ADRDA criteria for probable AD.	AMD	Retinal photos reviewed by experienced grader from Centre for Eye Research plus categorized as AMD based on software
Hamedani, 2019 [25]	USA	Cross-sectional	Not described	Not described	Community setting. Medicare database includes 97% of those aged 65+ in US (47,582,342 beneficiaries). Highly representative.	Not described	ICD-9 in claims data	Blindness or low vision	Blindness/low vision was defined by ICD-9 diagnosis codes (369.0–369.4) in claims data.
Heun, 2013 [26]	UK	Case-control	Mean 85.1 ± 8.2;	Male 34.9% Female 65.1%	Based on hospital register so likely included all patients meeting criteria during a defined period but unclear. Low representativeness.	634	ICD-10 codes in hospital discharge data	Glaucoma	ICD-10 codes in hospital discharge data
John, 1999 [27]	UK	Case-control	Mean 84.7 (Range 73.6–96.4)	Male 33.3% Female 66.7%	Community setting. Sample of 500 older persons from Oxfordshire in the UK. Low representativeness.	79	Port-mortem diagnosis established by consortium.	Cataract	Based on medical records from annual physical and neurological assessments
Kang, 2012 [28]	USA	Cross-sectional	≥ 60	Male 35.3%; Female 64.7%	17 Nursing homes in Iowa. 10 participants from each NH randomly selected from list of residents with dementia. Refusal rate of 10%. Highly representative of nursing home setting.	153	Diagnosis of AD or other dementia in medical chart	VI	Assessed by MDS section D1 Vision questionnaire.
Kiely, 2018 [29]	Australia	Cross-sectional	≥65; range 73 to 79; Mean: 75.3 ± 1.5	Male 62.5% Female 37.5%	Sampled from wave four of the oldest cohort of the PATH study, a representative community-based longitudinal cohort commencing in 2001 with follow-up every four years. Highly representative.	64	DSM-IV criteria for dementia or 5th Edition criteria for major neurocognitive disorder	VI	Impaired VA defined as > 0.3 logMAR (worse than 20/40 or 6/12) as assessed by a trained interviewer
Kosse, 2015 [30]	Netherlands	Cross-sectional	Not described	Male 60% Female 40%	Residents living in a 20-bed closed psychogeriatric ward in nursing home between September 2011 and April 2013. Low representativeness.	20	Extracted from electronic medical records.	Visual problems	Extracted from electronic medical records.
Lai, 2017 [31]	Taiwan	Case-control	≥45; Mean 78.7 ± 6.6	Male 42.6% Female 57.4%	Database of NHI. People 65+ with ICD-9 diagnosis of AD in 2000–2011 + 4 controls for each AD case. Highly representative as most people in Taiwan are covered by the NHI.	1351	ICD-9 codes listed on insurance claims for 2 or more visits	Glaucoma	ICE-9 codes in medical claims data for 2 or more visits.
Lawrence, 2009 [9]	UK	Qualitative	65–99	Male 36.8% Female 63.2%	Participants drawn from 4 socially and ethnically diverse south London boroughs. Highly representative.	19	Using a dementia service OR using a vision service + the “MMBlind”, the “Short Form of the Informant Questionnaire of Cognitive Decline in the Elderly” and the CDR scale	VI	Assessed with Snellen acuity and Seeing Severity Scale.
Löppönen, 2004 [32]	Finland	Cross-sectional	Mean 82.4 ± 7	Male 32% Female 68%	Community sample. 12% of the population in Lieto was invited. Participation rate of 82%. Highly representative.	112	Clinical assessment and DSM-IV + NINDS-AIREN for vascular dementia	Cataract Glaucoma	ICD-10 code in medical records.
Luo, 2018 [33]	China	Cross-sectional	Not described	Male 38.6% Female 52.5%	Data from Second National Samples Survey on Disability from April to May 31,2006. Covers all provincial administrative areas in Mainland China. Highly representative.	1208	Combination of self-report and on-site diagnosis by psychiatrist according to ICD-10.	VI	Used WHO criteria but limited to VI due to uncorrectable causes. Assessed by ophthalmologist.
Marquie, 2019 [34]	Spain	Cross-sectional	Mean age 81.4 ± 7.2.	Male 31.6% Female 69.4%	Recruited from public memory clinic; program that assesses cognition in community for free without referral; and a cohort study. Response rate 96.3%. Moderately representative.	833	Clinical diagnosis based on DSM-IV	Glaucoma AMD Low VA High IOP	Based on examination by an optometrist
Morse, 2004 [35]	USA	Cross-sectional	Mean: 84	Not described	Randomly selected from 11 New York city long-term-care facilities. Highly representative.	391	Not described	VI	Normal VA = 20/20–20/40; mild VI 20/50–20/70; moderate VI 20/80–20/200; severe VI 20/250–20/1000, very severe VI = counting fingers, hand motion, or no light perception. Assessed by Vistech Consultant.
Muurinen, 2014 [36]	Finland	Cross-sectional	> 65; Mean: 83	Male 22% Female 78%	All permanent residents in assisted living facilities in two cities in 2007. 70% participation rate. Moderately representative.	833 (1398)	Not described	VI	Based on answer to question “Is the resident’s vision good enough for reading regular print” yes/no (with or without glasses). Response of no = VI. Reported from trained nurses who knew the residents well.
Nyman, 2017 [37]	UK	Qualitative	Mean: 82.1; Range: 58–96	Male 34.6% Female 65.4%	Not described	26	Has received a formal diagnosis of dementia or has been referred for/in the process of receiving dementia assessment.	VI	Certified as having VI, registered blind or partially sighted, or self-reported low vision.
Patel, 2019 [38]	USA	Cross-sectional	≥ 65	Not described	Recruited from 2011 to 2016 from the NHATS, a nationally representative survey of 11,558 Medicare enrollees age ≥ 65. Highly representative.	Not described	Unclear but based on tests of memory, orientation and executive function	VI	Difficulty recognizing someone across the street. Self- reported with no further details.
Pelletier, 2014 [39]	Canada	Case-control	Mean 83.7 ± 6.3; Range: 66–101	Male 29.6% Female 71.4%	Recruited people with dementia from 2 academic hospitals, admitted from April 2008 to April 2009. Highly representative.	220	Clinical diagnosis with DSM-IV criteria. Had to have received diagnosis either before or during admission.	Glaucoma	Based on medical records or use of medication
Prince, 2011 [40]	China, India, Cuba, Dominican Republic, Venezuela, Mexico, Peru	Cross-sectional	≥ 65	More females than males in all sites	All residents aged 65+ in 11 geographically defined sites in seven LAMIC (India, China, Cuba, Dominican Republic, Venezuela, Mexico and Peru). Highly representative.	Not described	Diagnosis based on meeting either 10/66 or DSM-IV criteria.	VI	Eyesight problems which result in at least some difficulty, and/or an observer-rated item by the interviewer of ‘near total blindness’
Smilnak, 2019 [41]	USA	Case-control	≥ 75 Mean age 88.6 ± 5.9	Male 33.9% Female 66.1%	Pathologic specimens of eyes and brains of autopsy subjects aged 75 and above who presented to Duke University Medical Center. Low representativeness as only includes those who have died.	115	Autopsy and pathological diagnosis	AMD Glaucoma (severe)	Autopsy and histopathological diagnosis.
Tamura, 2006 [42]	Japan	Case- control	Mean: 80.9 ± 8.4	Male 17.2% Female 82.8%	Institutionalized residents or those accessing treatment at 4 hospitals. Highly representative.	172	Diagnosis of probable AD was based on clinical findings according to NINCDS-ADRDA	Glaucoma	Probable OAG was diagnosed by width of the angle of the anterior chamber >grade 2, a vertical cup-to-disc ratio of the optic nerve head > 0.7 and/or difference between the vertical cup-to-disc ratio in the eyes > 0.2 with characteristic glaucomatous disc change. Ophthalmic examination was performed and diagnosis was made by two glaucoma specialists
Varadaraj, 2020 [43]	USA	Cross-sectional	> 65;	Not described	Recruited form the NHATS, a nationally representative survey of Medicare beneficiaries aged 65 years and older. Highly representative.	Not described	Diagnosis of probable dementia based on (a) participant- or proxy-reported physician diagnosis of dementia or AD, or (b) an AD8 score ≥ 2; or (c) participant cognitive test scores ≤1.5 SDs below mean in at least two of the three cognitive domains. Possible dementia based on cognitive test scores ≤1.5 SDs below mean in one domain in the absence of meeting the physician diagnosis or AD8 criteria described above.	VI	Vision impairment was defined as self-reported blindness or difficulty with distance/near vision
Williams, 2014 [44]	UK	Case-control	Mean 80.1 ± 7.7	Male 36.8% Female 63.2%	Opportunistic rather than consecutive recruitment of cases to a primary care clinic. Low representativeness.	258	Clinical examination and NINCDS criteria	AMD	Based on photos and Wisconsin AMD grading system.
Wittich, 2019 [45]	Canada	Cross-sectional	Not described	Not described	Not described	21	Clinical diagnosis by consensus. Specific clinical criteria used are not described.	Reduced visual acuity	Reduced reading acuity (MNRead) (> .5 logMAR [20/63]). Moderate to severe loss of contrast sensitivity (Mars test) (< 1.48 log CS [3.3% contrast]).
Wong, 2015 [46]	Singapore	Cross-sectional	> 60	Not described	Recruited from 3 tertiary hospitals. Consecutive recruitment from July 2009 to December 2012. Moderately representative.	268 (outcome data on only 264)	DSM-IV criteria	AMD Diabetic retinopathy Cataract Glaucoma	Retinal photographs reviewed by ophthalmologist

*Abbreviations*: *AD* Alzheimer’s diseases, *AIBL* Australian Imaging Biomarkers and Lifestyle Study of Ageing, *AMD* Aged-macular degeneration, *CDR* Scale, Clinical Dementia Rating scale, *CFAS* Cognitive Function and Ageing Studies, *CS* Contrast sensitivity, *DSM-IV* he Diagnostic and Statistical Manual of Mental Disorders, 4th Edition, *ICD-10* The International Classification of Diseases, Tenth Revision, *ICD-9* The International Classification of Diseases-9, *ICD-9-CM* International Classification of Diseases, Ninth Revision, Clinical Modification, *IOP* Intraocular pressure, *LogMAR* Logarithmic minimum angle of resolution, *MCBS* The Medicare Current Beneficiary Survey, *MDS* The Minimum Data Set, *N* Number, *NH* Nursing home, *NHATS* National Health and Aging Trends Study, *NHATS* The National Health and Aging Trends Study, *NHI* National Health Insurance, NHS, National Health Service, *NINCDS* National Institute of Neurological and Communicative Disorders and Stroke, *NINCDS-ADRDA* National Institute of Neurological and Communicative Disorders and Stroke and the Alzheimer’s Disease and Related Disorders Association, *NINDS-AIREN* National Institute of Neurological Disorders and Stroke and Association Internationale pour la Recherché et l’Enseignement en Neurosciences, *OAG* Open-Angle Glaucoma, *AD8* Eight-item Informant Interview to Differentiate Aging and Dementia, *SDs* Standard deviations, *PATH study* The Population Assessment of Tobacco and Health Study, UK The United Kingdom, *US* The United States, *USA* The United States, *VA* Visual acuity, *VI* Visual impairment, *WHO* World Health Organization

^a^Standard deviation of 123 reported in the paper but is likely a typographical error

Among the prevalence studies, 18 (58%) studies were cross-sectional and 12 (39%) were case-control studies. Sixteen studies reported the prevalence of general VI. General VI was measured in different ways across studies including self-report [13, 23, 28, 36, 40], medical records [18, 19, 21, 22, 25–27, 30–32, 39, 41]or ophthalmologist assessment [14, 15, 17, 20, 24, 29, 33–35, 42, 44–46]. Fourteen studies [14, 15, 17–19, 21, 26, 31, 32, 34, 39, 41, 42, 46] reported the prevalence of glaucoma in various settings. Seven studies [17, 18, 24, 34, 41, 44, 46] reported the prevalence of aged-related macular degeneration (AMD). There was substantial variation among the seven studies in how the presence of AMD was determined. Three studies [17, 18, 34] measured the presence of AMD by optometric eye examination (prevalence 5 to 23%), three [24, 44, 46] studies through review of retinal photography by an experienced ophthalmologist (prevalence 17 to 41%), and one [41] study by autopsy and pathological diagnosis (prevalence of 53%). Only six studies [12, 17, 19, 27, 32, 46] reported the prevalence of cataract in which two studies were cross-sectional studies and reported a prevalence of cataract of 59% [17] and 21% [32].

Among the studies on the impact of VI, five (50%) studies were cross-sectional, two were cohort studies (20%) and three (30%) were qualitative studies. Lawrence et al. [47] published three papers (in 2009, 2010 and 2011) which presented results from one individual study. Bowen et al. [17] carried out two separate studies on different samples. One was a cross-sectional quantitative study on a large sample while the other one was a qualitative study on a small number of participants. Two studies were on the impact of VI on caregivers, in which one was a cross-sectional quantitative study [13] and the other one was a qualitative study [3]. Five of the quantitative studies were cross-sectional [17, 28, 29, 38, 40] and only two were longitudinal [16, 23]. Two of the qualitative studies [37, 47] only included participants with both dementia and VI, with no comparison with those living with dementia alone.

### Prevalence

Thirty-one studies provided data on the prevalence of VI or specific eye diseases in older people living with dementia in various settings (Table 2). Sixteen studies reported the prevalence of general VI with prevalence rates ranging from 0.4 to 52% [12, 13, 17, 19, 20, 22, 23, 25, 28–30, 33, 35, 36, 40, 45].

**Table 2:** Prevalence of visual impairment in people with dementia in the community, nursing homes, and hospitals or other healthcare settings

Study and Year	Setting	Age (years)	Definition of VI or eye disease	Method of measurement of VI or eye disease	Prevalence (%)
**Visual impairment**					
Abdullah, 1965^22a^	Community	≥ 65	Ability to read or not	Not described	Patient in long-stay ward: 33.76% Patient seen in domiciliary visit: 12.4%
Amjad, 2019 [13]	Community	≥65; Mean: 82.3 ± 8.1	Categorised based on responses to the following: ‘Uses corrective lenses or blind’, ‘Sees well enough to recognize person across street’, ‘Can watch TV across room’, ‘Reads newspaper print’. Presence of hearing or vision impairment measured yes/no Sensory symptom burden score range 0–8. Deaf or blind = 4 points. Each difficulty = 1 point	Self-reported	About 34%
Bowen, 2016 [17]	Community	60–89	Visual acuity worse than 6/12 or worse than 6/18 measured before and after refraction.	Ophthalmologist assessment	With spectacles and VA < 6/12: 32.5% With spectacles and VA < 6/18: 16.3%
Chandra, 1986 [19]	Community	Mean: 80.1	Listed as a cause of death	Death certificate	Blindness: 0.4%
Chriqui, 2017 [20]	Nursing home	≥ 65; Mean: 87.2 ± 7.5	A distance VA lower than 6/12 (0.30 logMAR, 20/40) in the better seeing eye	Ophthalmologist assessment	37.3% (95% CI, 29.1 to 46.1)
Deardorff, 2019 [23]	Community	≥65	Based on the question: How much trouble do you have with your vision? (no trouble, little trouble, or a lot of trouble). Subjects who reported “little trouble” or “a lot of trouble” were classified as visually impaired	Self-reported	45.9%
Kang, 2012 [28]	Nursing home	≥ 60	MDS-Activities of Daily Living (MDS-ADLs). Visual function was assessed by MDS section D1 Vision, which is a five-level ordinal variable scale (i.e., adequate, moderately impaired, impaired, highly impaired, and severely impaired). “Vision” refers to the ability to see in adequate light and with glasses.	Self-reported	Impaired: 22.2% Moderately impaired: 7.2% Highly impaired: 10.5% Severely impaired:1.3% In total: 41.2%
Kiely, 2018 [29]	Community	≥65; Mean: 75.3 ± 1.5	Sensory loss was defined by visual acuity worse the 0.3 logMAR (6/12 or 20/40)	Ophthalmologist assessment	15.4%
Luo, 2018 [33]	Community	Not described	WHO best–corrected visual acuity (BCVA) criteria (low vision: 0.05 ≤ BCVA ≤0.29; blindness: no light perception ≤ BCVA < 0.05, visual field less than 10 degrees; the better- seeing eye).	Ophthalmologist assessment	16.4%
Marquie, 2019 [34]	Community	Mean: 81.4 ± 7.2	Reduced visual acuity was defined as a standard LogMAR fraction scale ≤20/50 at 20 ft. (equivalent to a fraction scale of 6/15 at 6 m and a decimal scale of 0.4) according to the Snellen scale	Ophthalmologist assessment	Low VA: 37%
Morse, 2004 [35]	Nursing home	Mean: 84	VA was grouped into five categories: normal VI (20/20–20/40), mild VI (20/50–27,170), moderate VI (20/80–20/200), severe VI (20/250 20/1000), and very severe VI (counting fingers, hand motion, or no light perception).	Ophthalmologist assessment	Mild VI: 16.9% Moderate VI: 22.8% Severe VI: 11.8% Very severe VI: 0.2% In total: 51.6%
Muurine, 2014 [36]	Nursing home	> 65; Mean: 83	Residents’ vision was assessed by a question “Is the resident’s vision good enough for reading regular print” (yes/no) (with or without glasses). Those responding “no” were defined as visually impaired.	Self-reported	19.7%
Prince, 2011 [40]	Community	≥ 65	Eyesight problems which result in at least some difficulty, and/or an observer-rated item by the interviewer of ‘near total blindness’	Self-reported/observer-rated	**Latin America** Questionable dementia 42.3% Mild dementia 45.5% Moderate or severe dementia 39.6% **India** Questionable dementia 21.6% Mild dementia 16.2% Moderate or severe dementia 0% **China** Questionable dementia 12.9% Mild dementia 16.1% Moderate or severe dementia 20.9%
Clague, 2017 [22]	Community	Mean: 82.6 ± 7.4;	Identified open angle glaucoma cases by the principal diagnosis of ICD-9-CM codes 365.1, 365.10, or 365.11	Medical claim during ambulatory care visits	Blindness/low vision 4.0%
Hamedani, 2019 [25]	Community	Not described	Blindness/low vision was defined by ICD-9 diagnosis codes (369.0–369.4).	Claims data	Blindness/low vision AD: 23.9% Dementia NOS: 50.0%
Kosse, 2015 [30]	Nursing home	Not described	Categorized according to the anatomical therapeutic chemical (ATC) classification system	Medical records	Visual problems: 45%
Wittich, 2019 [45]	Not described	Not described	Reduced reading acuity: > .5 logMAR [20/63]	Ophthalmologist assessment	Reduced VA: 23.80%
**AMD**					
Bowen, 2016 [17]	Community	60–89	AMD was classified into dry and wet (neovascular) AMD and then graded as mild, moderate or severe.	Ophthalmologist assessment and medical records	17.7%
Carcenac, 2019 [18]	Nursing home	Not described	Not described	Medical records	Reported in records: 13.6% Discovered in eye exam: 23.1%
Frost, 2016 [31]	Community	Mean: 70.2 ± 9.0	Classified according to the Beckman system: No clinically relevant signs of AMD: Small drusen or no drusen, no pigmentary abnormalities. Early AMD: Medium drusen without pigmentary abnormalities. Intermediate AMD: Large drusen, or Medium drusen with pigmentary abnormalities Late AMD: Lesions associated with neovascular AMD or geographic atrophy	Ophthalmologist assessment	Early AMD: 36% Intermediate AMD: 4.5% Late AMD: 0 Total AMD: 40.9%
Marquie, 2019 [34]	Community	Mean: 81.4 ± 7.2	Diagnoses of dry and wet AMD were based on the Age-Macular Degeneration Preferred Practice Patterns guidelines; used the classification of the Age-Related Eye Disease Study and a more recent classification to define the early and intermediate stages of AMD	Ophthalmologist assessment	4.8%
Smilnak, 2019 [41]	Hospital	≥ 75; Mean: 88.6 ± 5.9	AMD was defined as Sarks grades III-VI, corresponding to intermediate to severe clinical AMD	Autopsy	53%
Williams, 2014 [44]	Other healthcare setting	Mean: 80.1 ± 7.7	The AMD grading system was as the “Whitla grades”	Ophthalmologist assessment	Grade 0: 47.3% Grade 1: 0.9% Grade 2: 7.8% Grade 3: 8.1% Ungraded: 19.4%
Wong, 2015 [46]	Not described	> 60	Assessed AMD using standard grading systems (not described)	Ophthalmologist assessment	27%
**Cataract**					
Abdullah, 1965 [12]	Community	≥ 65	Not described	Not described	48%
Bowen, 2016 [17]	Community	60–89	Cataract sufficient to be graded on the TOC cataract grading scale.	Ophthalmologist assessment and medical records	59%
Chandra, 1986 [19]	Community	Mean: 80.1	Not described	Medical records	0.2%
John, 1999 [27]	Community	Mean 84.7 (73.6–96.4)	Not described	Medical records	22.8%
Löppönen, 2004 [32]	Community	Mean 82.4 ± 7	Based on ICD-10 codes	Medical records	21%
Wong, 2015 [46]	Not described	> 60	Assessed using standard grading systems (not described)	Ophthalmologist assessment	74%
**Diabetic retinopathy**					
Bowen, 2016 [17]	Community	60–89	Not described	Ophthalmologist assessment	2%
Wong, 2015 [46]	Not described	> 60	Assessed using standard grading systems (not described)	Ophthalmologist assessment	19%
**Glaucoma**					
Bayer, 2002 [14]	Nursing home	With glaucoma; Mean: 72.9 ± 10.6 Without glaucoma Mean: 71.4 ± 11.9	The diagnosis of probable glaucoma required at least one of the following two criteria: - A characteristic pattern of glaucomatous visual field loss; A cup-to-disk ratios of 0.8 or greater with an optic nerve head appearance consistent with glaucoma.	Ophthalmologist assessment	25.9%
Bayer, 2002 [15]	Nursing home	With glaucoma: Mean: 71.9 ± 11.6 Without glaucoma: Mean: 73.2 ± 12.3^a^	The diagnosis of probable glaucoma required at least one of the following two criteria: - A characteristic pattern of glaucomatous visual field loss; - A cup-to-disk ratios of 0.8 or greater with an optic nerve head appearance consistent with glaucoma.	Ophthalmologist assessment	24.5%
Bowen, 2016 [17]	Community	60–89	Not described	Ophthalmologist assessment	7.1%
Carcenac, 2019 [18]	Nursing home	Not described	Not described	Medical records	Reported in records: 12.7% Discovered in eye exam: 22.2%
Chandra, 1986 [19]	Community	Mean: 80.1	Not described	Medical records	0.2%
Chung, 2015 [21]	Community	≥45; Mean: 76.8 ± 9.6	Based on ICD-9-CM codes 365.1, 365.10, or 365.11	Medical records	2.02%
Heun, 2013 [26]	Hospital	Mean: 85.1 ± 8.2;	Based on ICD-10 codes	Medical records	1.1%
Lai, 2017 [31]	Community	≥45; Mean: 78.7 ± 6.6	Based on ICD-9 codes 365.1 and 365.2	Medical records	7.9%
Löppönen, 2004 [32]	Community	Mean 82.4 ± 7	Based on ICD-10 codes	Medical records	6%
Marquie, 2019 [34]	Community	Mean: 81.4 ± 7.2	The glaucoma category was based on the image of the head of the optic nerve (ONH), ONH OCT findings and Icare IOP measurements; High IOP was defined as ≥24 mmHg using Icare Tonometry	Ophthalmologist assessment	Glaucoma: 7.7% High IOP: 6.5%
Pelletier, 2014 [39]	Hospital	Mean: 83.7 ± 6.3;	Not described	Medical records	9.5%
Smilnak, 2019 [41]	Hospital	≥ 75; Mean: 88.6 ± 5.9	The histopathologic diagnosis of advanced glaucoma was made when the following were observed: sparse retinal ganglion cells, diminished size of optic nerve axon bundles, and fibrotic thickening or “cupping” of the optic nerve	Medical records	Glaucoma (severe): 13.9%
Tamura, 2006 [42]	Nursing home	Mean: 80.9 ± 8.4	Probable OAG was diagnosed by width of the angle of the anterior chamber >grade 2, a vertical cup-to-disc ratio of the optic nerve head > 0.7 and/or difference between the vertical cup-to-disc ratio in the eyes > 0.2 with characteristic glaucomatous disc change.	Ophthalmologist assessment	23.8%
Wong, 2015 [46]	Not described	> 60	Optic disc features of glaucoma	Ophthalmologist review of retinal photographs	15%

*Abbreviation*: *VA* Visual acuity, *USA* The United States, *UK* The United Kingdom, *CI* Confidence interval, *VI* Visual impairment, *AD* Alzheimer’s diseases, *Dementia NOS* Dementia (not otherwise specified), *AMD* Aged-macular degeneration, IOP Intraocular pressure

^a^Authors reported this as incidence of cataract but based on the methods described it is most likely an estimate of prevalence

Fourteen studies [14, 15, 17–19, 21, 26, 31, 32, 34, 39, 41, 42, 46] reported the prevalence of glaucoma in various settings with prevalence ranging from 0.2 to 26%. Prevalence was substantially higher in studies in nursing homes [14, 15, 18, 42]. Seven studies [17, 18, 24, 34, 41, 44, 46] reported the prevalence of aged-related macular degeneration (AMD) in various settings, which ranged from 5 to 53%. Only six studies [12, 17, 19, 27, 32, 46] reported the prevalence of cataract with prevalence ranging from 0.2 to 74%. As well as variation in settings, there was variation in how the presence of cataract was assessed including an eye exam by an optometrist (prevalence of 59%) [17], review of medical records (prevalence of 23 and 21%) [27], review of retinal photographs by an ophthalmologist (prevalence of 74%) [46], and review of death certificates (prevalence of 0.2%) [18]. As death certificates only list major diseases, some minor diseases such as cataract may be missed leading to the low prevalence of VI when using this method to measure the presence of cataract. The prevalence of diabetic retinopathy was reported in only two studies [17, 46], with a prevalence of 19% in a hospital-based study in Singapore [46] and 2% in a community-based study in the UK [17]. In addition to prevalence, Bowen et al. also commented on the proportion of VI or eye diseases that were potentially reversible [17]. They found that much VI was due to refractive error that could be remediated by corrective lenses and eye diseases such as cataract that are amenable to surgical correction [17].

### Impact of VI in older people living with dementia

Ten studies (seven quantitative and three qualitative) provided data on the impact of VI in older people living with dementia (Table 3 and Table 4). There were a range of impacts examined in the studies including use of hospital services**,** level of disability and dependency, social engagement, negative emotions, abnormal behaviors, loss of hobbies, difficulty in using visual or memory aids, and Neuropsychiatric Inventory (NPI) symptoms.

**Table 3:** Impact of visual impairment on older people with dementia and their carers (Quantitative study)

Study and Year	Study type	Type of impact	Reference group	Comparison group(s)	Effect measure	Size of effect	Confidence interval/ ***P*** value
**Impact on older people with dementia and VI**							
Bennett, 2018 [16]	Cohort	Inpatient visit	VI and no dementia	VI and dementia	OR	3.5 (CFA I)	1.1–11.5
						1.7 (CFA II)	0.8–3.2
		Care workers’ service	VI and no dementia	VI and dementia	OR	5.8 (CFA I)	1.8–19.2
						6.4 (CFA II)	2.6–15.5
		Home care assistance	VI and no dementia	VI and dementia	OR	4.4 (CFA I)	1.3–15.0
						3.4 (CFA II)	1.3–8.5
Bowen, 2016 [17]	Cross-sectional	Activity of Daily Living impairment: Toilet/commode	No VI or dementia	VI and dementia	OR	2.19 (VA < 6/12)	1.35–3.53
						1.28 (VA < 6/18)	0.71–2.28
		Eating	No VI or dementia	VI and dementia	OR	1.75 (VA < 6/12)	1.06–2.90
						1.96 (VA < 6/18)	1.03–3.82
		Dressing	No VI or dementia	VI and dementia	OR	1.66 (VA < 6/12)	1.11–2.49
						1.2 (VA < 6/18)	0.71–2.00
		Hygiene	No VI or dementia	VI and dementia	OR	1.70 (VA < 6/12)	1.13–2.54
						1.46 (VA < 6/18)	0.86–2.45
		Teeth	No VI or dementia	VI and dementia	OR	1.86 (VA < 6/12)	1.22–2.84
						1.70 (VA < 6/18)	0.99–2.9
		Telephone	No VI or dementia	VI and dementia	OR	1.89 (VA < 6/12)	1.26–2.85
						2.08 (VA < 6/18)	1.22–3.56
		Shopping	No VI or dementia	VI and dementia	OR	1.63 (VA < 6/12)	1.03–2.61
						1.94 (VA < 6/18)	1.07–3.54
		Finances	No VI or dementia	VI and dementia	OR	1.7 (VA < 6/12)	1.09–2.66
						1.87 (VA < 6/18)	1.06–3.30
		Transport	No VI or dementia	VI and dementia	OR	1.91 (VA < 6/12)	1.14–3.24
						2.55 (VA < 6/18)	1.29–5.08
Deardorff, 2019 [23]	Cohort	Inpatient Admission	Dementia alone	Dementia + VI	OR	1.82	1.17–2.82
			Dementia alone	Dementia +HI	OR	1.03	0.80–1.52
			Dementia alone	Dementia + VI + HI	OR	1.39	0.96–2.01
		Hospice Use	Dementia alone	Dementia + VI	OR	1.18	0.50–2.76
			Dementia alone	Dementia +HI	OR	1.39	0.65–3.01
			Dementia alone	Dementia + VI + HI	OR	2.11	1.05–4.21
		Average Annual Total Healthcare Cost	Dementia alone	Dementia + VI alone	MD	10,466 (REF) 11,671 (Comparison group)	4600–23,812 9524–14,233 (*P* = 0.282)
			Dementia alone	Dementia + VI + HI	MD	10,466 (REF) 11,303 (Comparison group)	4600–23,812 9524–13,291 (*P* = 0.395)
		Average Annual Medicare Fee-for-Service Cost	Dementia alone	Dementia + VI alone	MD	3338 (REF) 3872 (Comparison group)	851–13,097 2804–5374 (*P* = 0.364)
			Dementia alone	Dementia + VI + HI	MD	3338 (REF) 3505 (Comparison group)	851–13,097 2637–4606 (*P* = 0.737)
Kang, 2012 [28]	Cross-sectional	Social engagement	Dementia alone	Dementia + VI	Beta coefficient	β = −0.167	*P* = 0.039^a^
Kiely, 2018 [29]	Cross-sectional analysis of longitudinal cohort study	NPI symptoms	No VI or dementia	Dementia alone	IRR	1.63	0.73–3.63
				Dementia + VI	IRR	7.08	1.41–35.43
				Dementia + Dual sensory loss	IRR	6.09	1.43–26.02
Patel, 2019 [38]	Cross-sectional	Activity Limitations	No VI or dementia	Possible dementia + VI	Predicted number of activity limitations:	1.97	01.72–2.21
				Probable dementia + VI	Predicted number of activity limitations:	2.28	2.03–2.53
Prince, 2011 [40]	Cross-sectional	Disability	Dementia alone	Dementia + VI	MD (WHODAS 2.0 scores)	Latin America: 6.5	+ 3.3 to+ 0.6
						China: −1.7	−12.5 to+ 9.12
						India + 12.3	+ 5.4 to + 19.3
**Impact on carers**							
Varadaraj, 2020 [43]	Cross-sectional	**Impact on valued activities**					
		Visiting friends and family	Dementia + VI	No VI or dementia	Percentage	Reference: 36.5% Comparison: 12.6%	*P* < 0.001
		Going out for enjoyment	Dementia + VI	No VI or dementia	Percentage	Reference: 31.3% Comparison: 7.3%	P < 0.001
		Attending religious services	Dementia + VI	No VI or dementia	Percentage	Reference: 14.5% Comparison: 5.4%	P < 0.001
		Participating in club meetings or group activities	Dementia + VI	No VI or dementia	Percentage	Reference: 15.3% Comparison: 5.2%	P < 0.001
		**Supportive services directed to carers**					
		Respite care	Dementia + VI	No VI or dementia	Percentage	Reference: 18.6% Comparison: 7.2%	P < 0.001
		Use of ≥1 supportive care	Dementia + VI	No VI or dementia	Percentage	Reference: 29.5% Comparison: 13.1%	P < 0.001
		**Caregiving hours per month**	Dementia + VI	No VI or dementia	IRR	1.7	1.4–2.2
		**Number of valued activities affected due to providing care**	Dementia + VI	No VI or dementia	IRR	3.2	2.2–4.6

*Abbreviations*: *CFAS* Cognitive Function and Ageing Studies, *HI* Hearing impairment, *IRR* Incidence Rate Ratio, *NCDS* Neurocognitive disorders (NCDs), *NPI* Neuropsychiatric Inventory, *OR* Odds Ratio, *P* P value, *REF* Reference group, *VA* Visual acuity, *VI* Visual impairment, *WHODAS 2.0* WHODAS, World Health Organization Disability Assessment Schedule version 2.0, *MD* Mean difference

^a^after adjustment for other covariates including Activity of Daily Life impairment, cognitive impairment, depression, anxiolytics and behavioral symptoms

**Table 4:** Impact of visual impairment on older people with dementia and their carers (Qualitative studies)

Study and Year	Age (years)	Sex	N with dementia	Type of impact	Impact
**Impact on older people with dementia and VI**					
Bowen, 2016 [17]	60–89	Male 50%; Female 50%	36	Negative emotions	➢ Feel awful ➢ Feel anxious
				Loss of hobbies	➢ Lose hobbies, cannot read books ➢ Cannot do word puzzles anymore
Lawrence, 2009 [9]	65–99	Male 36.8% Female 63.2%	19	Decreased independence	➢ Unable to locate themselves using visual cues ➢ Lose the ability to manage independently ➢ Reduced ability of older adults to perform certain activities safely ➢ Impaired ability to assess risks accurately ➢ Unable to navigate their surroundings as they can’t observe or recall the layout
				Negative emotions	➢ Feel distress ➢ Profound disorientation and distress can manifest as disruptive, agitated or aggressive behavior ➢ Older adults likely to adopt self-protective strategies, such as denial ➢ Experience loneliness and isolation.
				Decreased social engagement	➢ Restricted interests and hobbies and require other’s stimulation ➢ Difficult to participate in groups and often need one-to-one interaction ➢ Concerns about safety prompted family members to limit their relatives’ activities ➢ Difficult to identify when conversation was directed at them. Easier to interact on a one-to-one basis
Nyman, 2017 [37]	Mean: 82.1; Range: 58–96	Male 34.6% Female 65.4%	26	Decreased independence	➢ Presence of dementia and VI exacerbates existing difficulties leading to greater dependence ➢ Ability to use vision deteriorating and has to rely more and more on memory for independence. ➢ Some memory aids no longer usable because of VI as rely on vision to serve as reminder ➢ Some visual aids not usable as rely on short-term memory
				Negative emotions	➢ Boredom and lack of daily stimulation
				Loss of hobbies	➢ Can’t participate in scrabble ➢ Previously held hobbies abandoned
**Impact on carers**					
Lawrence, 2009 [9]	65–99	Male 37.8% Female 63.2%	19	Conflict	➢ Concerns about safety by carers lead to limitations on valued activities and roles. This creates conflict when the older person does not wish to give up these activities or roles.
				Exhaustion	➢ Caregivers physically exhausted - found it difficult to leave the older person for even brief periods of time. ➢ Increased emotional dependency of person with dementia as they are more socially isolated

*Abbreviations*: *N* Number, *VI* Visual impairment

Two cohort studies [16, 23] investigated increased use of hospital services by those with comorbid dementia and VI. Deardorff et al. [23] found that people with concurrent VI and dementia had a higher risk of inpatient admission when compared to older people living with dementia without VI or hearing impairment (HI) (Odds Ratio (OR) =1.82, 95% Confidence Interval (CI) = 1.17–2.82). However, there was no significant difference in odds of inpatient admission in older people living with dementia who had HI only or both VI and HI compared to those with no VI or HI. Hence, in this particular study the effect of VI was differentiated from the effect of HI and it appears that VI had an impact on hospital admission, whereas HI did not. There was also an increased likelihood of hospice use in those with comorbid visual and hearing impairment and dementia compared to those living with dementia alone (OR = activity limitation, 95%CI 1.05–4.21). But, there was no significant difference in older people living with dementia who had HI only or VI only compared to those with no VI or HI. Therefore, for this outcome it is difficult to differentiate between the effect of VI and HI but the effects do appear to be additive.

In regards to health costs, no differences were observed in total annual health costs and annual medical fee-for-service costs between those living with dementia with and without sensory impairment. Bennett et al. [16] found increased inpatient visits in those living with dementia and VI compared to those with VI alone (Cognitive Function and Ageing Study (CFAS))(CFAS I: OR = 3.5, 95%CI = 1.1–11.5; CFAS II: OR = 1.7, 95%CI = 0.9–3.2).

Decreased Activities of Daily Living (ADLs) was reported in three cross-sectional studies [17, 38, 47]. In a large study of community-dwelling people living with dementia in the UK, comorbid VI (defined as Visual acuity (VA) < 6/12) compared to dementia without VI was significantly associated with less independence in ADLs (*P* < 0.05) [17]. Patel et al. [38] found that people with possible dementia and VI had more activity limitation compared to those with no dementia and no sensory impairments (OR = 1.97, 95%CI 1.72–2.21) and that this was greater than the activity limitation observed in those with possible dementia alone compared to those with no dementia and no sensory impairments (OR = 1.24, 95%CI 1.14–1.33). Conversely, there did not seem to be a greater association of VI and probable dementia on activity limitations compared to probable dementia alone, which suggests that VI may not lead to any additional limitations on the activities of older people living with dementia.

A further three studies reported the impact of comorbid VI on increased dependence in older people living with dementia [16, 37, 47]. In a longitudinal study, Bennet et al. [16] found that use of care workers was about six times greater in older people living with dementia and VI in two samples (CFA I: OR = 5.8, 95%CI = 1.8–19.2; CFA II: OR = 6.4, 95% CI = 2.6–15.5) compared to those with VI without dementia. In addition, the use of home help in the previous 4 weeks before the interview was three to four times greater (CFA I: OR = 4.4, 95%CI = 1.3–15.0; CFA II: OR = 3.4, 95% CI = 1.3–8.5). A global study on the impact of VI in those living with dementia found a cross-sectional association with disability in some settings [40]. Participants living with dementia and VI in Latin America (Mean difference = 6.5, 95% CI = 3.3–9.6) and India (Mean difference = 12.3, 95% CI = 5.4–19.3) had a significantly higher score on the World Health Organization Disability Assessment Schedule than those living with dementia without VI.

Lawrence et al. [47] carried out a qualitative study in people with both dementia and VI and found that they experienced disorientation due to an inability to see the clock or read the date, increasing their dependency on others. Further, they were unable to compensate for poor memory using visual cues or compensate for poor vision with cognitive strategies, resulting in a greater impact of both conditions. Caregivers’ increased concerns over their safety meant that there were often increased restrictions placed on their activities. Another community-based qualitative study in the UK of people with VI and dementia also found greater dependence [37]. However, similar to the previous study, there was no comparison with people living with dementia alone.

The qualitative study by Lawrence et al. [47] also found that people with dementia and VI felt lonely and isolated. They suffered from difficulties in initiating social contact and identifying when conversation was directed at them. This added to the burden of engaging in group conversations, leading to decreased social engagement. A South Korean cross-sectional study [28] also found a statistically significant association between VI and reduced social engagement (*P* = 0.021) in a group of older people living with dementia.

A negative emotional impact was reported in two qualitative studies [37, 47]. Nyman et al. [37] found that older people living with dementia and VI felt bored and lacked daily stimulation as reported by themselves and their caregivers. However, the study did not include any older people living with dementia alone to enable comparison of the added impact of VI. Lawrence et al. [47] found that older people living with dementia and VI who felt distressed sometimes manifested this distress as agitated and aggressive behavior. In a cross-sectional analysis, Kiely et al. [29] also found that VI was associated with greater NPI symptoms in those living with dementia (Incidence rate ratio (IRR) = 7.08, 95% CI = 1.41–35.43) compared to those without VI or dementia. Those living with dementia alone did not demonstrate significantly greater NPI symptoms (IRR = 1.63, 95%CI = 0.73–3.63).

The loss of hobbies is another important impact which was reported in two qualitative studies [17, 47] where respondents reflected on experiences before and after developing VI. For example, Bowen et al. [17] reported a caregiver who stated that his wife living with dementia had to give up her hobby of reading due to VI. Participants in the study by Lawrence et al. [47] also reported that joint VI and memory loss restricted interests and hobbies and meant that those living with dementia and VI needed to be stimulated by others.

### Impact of VI on caregivers of older people living with dementia

There were only two studies (one qualitative and one quantitative) that specifically investigated the added impact of VI to dementia on caregivers, both in a community setting [43, 47] (Tables 3 and 4). A UK qualitative study reported negative impacts on caregivers of those living with dementia and VI including negative emotions and loss of hobbies [17]. However, there was no comparison group of caregivers of those living with dementia alone. Caregivers also reported conflict when taking care of older people living with both dementia and VI due to concerns about safety resulting in greater restrictions of valued activities and roles of those living with dementia. Caregivers also reported physical exhaustion due to the high dependency level of those with dementia and preventing them from leaving the older person with dementia alone. The second study was a cross-sectional quantitative study comparing experiences of caregivers of older people living with dementia and VI, with dementia only, VI only or no dementia or VI. Results demonstrated that caregivers of older people living with concurrent dementia and VI had less time to participate in activities such as: visiting friends and family, going out for enjoyment, attending religious services, and participating in club meetings or group activities. Moreover, caregivers of people living with dementia and VI spent 1.7 times more hours on caregiving (95% CI = 1.4–2.2) than caregivers of those without either dementia or VI. In contrast caregivers of those living with dementia only spent 1.3 times more hours of caregiving (95% CI = 1.1–1.6) compared to caregivers of those with no dementia or VI.

## Discussion

In this scoping review we found that VI is common in older people living with dementia with the prevalence varying based on the setting and method of measurement of VI. Glaucoma, AMD, cataract and diabetic retinopathy were also common. Concurrent VI resulted in multiple different impacts on older people living with dementia and their caregivers. However, research on the impacts of concurrent VI on caregivers was quite limited.

Despite being able to locate a few studies reporting the prevalence of VI in older people living with dementia, a clear understanding of the prevalence of VI and common eye diseases in this population based on current research is not possible given the heterogeneity between studies in terms of setting, age of participants, and how VI and eye diseases were defined and measured. How VI is measured is a particularly important consideration, given that VI in older people living with dementia is likely to be under-diagnosed [17] with measurement based on of self-report, medical records, claims data and death certificates likely to underestimate prevalence. In addition, the use of definitions that include the use of glasses to define VI do not adequately capture the likely differential impact of having VI adequately corrected or not. The study by Bowen et al. [17] was the only study that carried out formal visual acuity assessments in a large cohort recruited from 20 National Health Service sites in six English regions. Participants from both a community and nursing home setting were included and the prevalence estimates of 32.5% (with spectacles and VA < 6/12) and 16.3% (with spectacles and VA < 6/18) are likely to be the most accurate estimates of prevalence of VI in older people living with dementia.

Many of the studies that provided data on the prevalence of different eye conditions were case-control studies of very small sample size that were primarily conducted to determine causal relationships between particular eye diseases and dementia rather than to estimate prevalence [14, 15, 24, 27]. In many of these studies it was unclear how representative the cases of dementia were and, as a result, the reported data are unlikely to provide an accurate estimate of prevalence. There was also surprisingly little data on the prevalence of cataract in those living with dementia and the studies that were conducted had different study designs, were in different settings and used different methods for assessing the presence of cataract resulting in a wide range of prevalence estimates (0.2 to 74%). Similar to VI, the best estimate of cataract prevalence is that of 59% (95%CI 55.2 to 62.7%) reported by Bowen et al. [17] which measured cataract with an eye exam by an optometrist in a representative sample of people living with dementia living in the community or nursing homes. Surprisingly only two studies reported on the prevalence of diabetic retinopathy among older people living with dementia [17, 46].

Our results demonstrate that despite the seemingly high prevalence of VI and eye diseases in older people living with dementia, there is a significant unmet need with limited research done to accurately estimate this prevalence. In particular, there is a need for research that includes assessments by optometrists and/or ophthalmologists to more accurately determine VI and eye diseases. Given that the study by Bowen et al. [17] found that much VI was due to refractive error that could be remediated by corrective lenses, or eye diseases such as cataract that are amenable to surgical correction, identifying the presence of these common and treatable eye conditions in older people living with dementia is of clear importance to public health. Preventable vision loss due to cataract (reversible with surgery) and refractive error (reversible with spectacle correction) continue to cause most cases of blindness and moderate or severe vision impairment in adults aged 50 years and older [48].

We found evidence of a range of negative impacts of VI in people living with dementia and two studies suggesting negative impacts on their caregivers. Similar to prevalence, we observed large heterogeneity in how VI and eye diseases were measured and defined and we would recommend that future studies of the impact of VI on those living with dementia should have a clear definition of VI, confirmed by a comprehensive visual assessment rather than being self-reported. There is also a need for more longitudinal studies as most studies examining the impact of comorbid VI on those living with dementia were cross-sectional, which makes it harder to establish a causal relationship. Moreover, several studies examining the impact of comorbid VI and dementia either had inappropriate or no comparators to adequately examine the additional impact of having VI in people living with dementia. For example, a longitudinal study found that use of care workers was six times greater in older people living with dementia and VI in two samples [16]. However, this was compared to those with VI without dementia rather than those living with dementia without VI. A few qualitative studies reported a range of negative impacts, including on ADL function, social isolation and psychological health. These qualitative studies only included older people living with concurrent dementia and VI and no comparison group. Hence, it is difficult to be sure what negative impacts are due to the added presence of VI and what impacts are the result of dementia itself.

There are also many potential impacts that have not been assessed, such as the impact of VI in those living with dementia on the risk of falls. Although both VI and dementia are known falls risks factors [49, 50], it is not known whether the combined presence of these two conditions greatly multiplies this risk. Our scoping review also found only a limited number of studies looking at the impact of concurrent VI and dementia on caregivers, with only one qualitative study and one quantitative study addressing this topic [43, 47]. Many studies reporting comments from caregivers were about the impact of VI on those living with dementia, rather than the impact of VI on themselves as part of their caring role. Therefore, an important area of further study would be to investigate the impact of VI on caregivers of those living with dementia, including consideration of health outcomes of caregivers. Impact on health service use and health expenditure is another key area that requires more research. Again, we found only one study that examined use of health and support services [17], but the lack of an appropriate comparator group prevented assessment of the added impact of VI on health service use in those living with dementia. Given population ageing, the number of people with concurrent dementia and VI will sharply grow over coming years, and assessment of the impact of these comorbid conditions on health expenditure and research into interventions aimed at reducing this expenditure by better management of VI in those living with dementia is crucial.

To our knowledge, this is the first scoping review to examine specifically the prevalence of VI in people living with dementia and the impact of VI on people living with dementia and their caregivers. We have identified important gaps in the evidence-base that should be addressed by future research. A limitation of our approach is that as we conducted a scoping review, we did not conduct a formal assessment of the quality of included studies. However, we did consider the representativeness and setting of studies, study design and definition of VI due to the enormous variation in these aspects between studies.

## Conclusions

In conclusion, VI is common in older people living with dementia and is associated with negative impacts on those living with dementia and their caregivers. We conducted a systematic search across a large number of electronic databases identifying important gaps in the literature., The heterogeneity between studies in terms of setting and method for assessing VI make it difficult to compare findings between studies. Research is limited, particularly in terms of impacts on caregivers and longitudinal research. This review emphasizes the importance of managing vision problems in older people living with dementia and provides directions for future targeted research on this relatively neglected topic.

## Supplementary Information

**Additional file 1.** Search strategy in OVID MEDLINE.

## Data Availability

All data generated or analyzed during this study are included in this published article (and its supplementary information files).

## Abbreviations

ADLs: Activities of Daily Living
AMD: Aged-related macular degeneration
CFAS: Cognitive Function and Ageing Study
CI: Confidence interval
IRR: Incidence rate ratio
NPI: Neuropsychiatric Inventory
OR: Odds Ratio
UK: The United Kingdom
US: The United States
VA: Visual acuity
VI: Visual impairment
