# Supplementary material for: Prevalence of visual impairment in older people living with dementia and its impact: a scoping review
Source: BMC Geriatr. 2023 Feb 1;23:63. doi: 10.1186/s12877-022-03581-8 (PMC9890816; doi:10.1186/s12877-022-03581-8)
Supplement: Supplementary file 1 — Additional file 1. Search strategy in OVID MEDLINE. [file 12877_2022_3581_MOESM1_ESM.docx]

Supplementary File. Search strategy in OVID MEDLINE

| **#** | **Search Statement** | **Results** |
| --- | --- | --- |
| 1 | exp eye disease/ | 559858 |
| 2 | ((sight or vision or visual) adj4 (impair* or disorder* or disabilit* or degenerate*)),mp, | 45569 |
| 3 | 1 or 2 | 570315 |
| 4 | exp dementia/ | 162875 |
| 5 | (dementia or frontotemporal lobar or lewy body or huntington). mp. | 139836 |
| 6 | 4 or 5 | 207830 |
| 7 | exp aged/ | 3076987 |
| 8 | (aged or elderly or old* people).mp. | 5350845 |
| 9 | 7 or 8 | 5350845 |
| 10 | 3 and 6 and 9 | 2019 |
